# Microbial Volatile Organic Compounds: An Alternative for Chemical Fertilizers in Sustainable Agriculture Development

**DOI:** 10.3390/microorganisms11010042

**Published:** 2022-12-22

**Authors:** Murugesan Chandrasekaran, Manivannan Paramasivan, Jesudass Joseph Sahayarayan

**Affiliations:** 1 Department of Food Science and Biotechnology, Sejong University, Neungdong-ro 209, Gwangjin-gu, Seoul 05006, Republic of Korea; 2 Department of Microbiology, Bharathidasan University, Tiruchirappalli 620024, Tamilnadu, India; 3 Department of Bioinformatics, Alagappa University, Karaikudi 630003, Tamilnadu, India

**Keywords:** food security, induced systemic resistance, microbial volatile organic compounds, soil fertility, sustainable agriculture

## Abstract

Microorganisms are exceptional at producing several volatile substances called microbial volatile organic compounds (mVOCs). The mVOCs allow the microorganism to communicate with other organisms via both inter and intracellular signaling pathways. Recent investigation has revealed that mVOCs are chemically very diverse and play vital roles in plant interactions and microbial communication. The mVOCs can also modify the plant’s physiological and hormonal pathways to augment plant growth and production. Moreover, mVOCs have been affirmed for effective alleviation of stresses, and also act as an elicitor of plant immunity. Thus, mVOCs act as an effective alternative to various chemical fertilizers and pesticides. The present review summarizes the recent findings about mVOCs and their roles in inter and intra-kingdoms interactions. Prospects for improving soil fertility, food safety, and security are affirmed for mVOCs application for sustainable agriculture.

## 1. Introduction

Microbial volatile organic compounds (mVOCs) are a type of volatile organic compound produced by microorganisms, especially bacteria and fungi, during their metabolism [1,2]. The mVOCs are designated as lipophilic compounds with a low boiling point, low molecular mass (an average of 300 Da), and high vapor pressure (0.01 kPa) [3,4]. These characteristics facilitate the evaporation and diffusion of mVOCs and their roles in plant growth and protection via pores in soil and rhizosphere environments. In addition, mVOCs act as an ideal signal/messenger molecule for mediating interactions at both short and long distances in microbes and plants [1,2,3,4,5,6]. The richness of mVOCs has been cataloged in the mVOCs 2.0 and 3.0 database [7,8]. Based on a literature survey, in 2014, the mVOCs 2.0 database comprised ~1000 volatiles emitted by 69 fungi and 349 bacteria [7], whereas, in 2018, the mVOCs 3.0 database contained 1860 unique mVOCs emitted from 604 bacterial and 340 fungal species [8]. Recent studies have also been strengthened based on their importance in food production, food safety, and eco-friendly, cost-effective, and sustainable approaches to help modern agriculture [1,2,3,4,5,6,7,8,9]. The mVOCs possess broad-spectrum bioactivities such as plant growth promotion [10], abiotic stress resistance [11,12,13], plant defense [14], insect-pest resistance [15], etc. There are various kinds of microbial interactions taking place belowground/aboveground such as bacteria–plant, fungi–plant, bacteria–bacteria, fungi–fungi, fungi–bacteria, bacteria–protists, and bacteria–fungi–plant interactions. Among microorganisms, *Bacillus subtilis* remain the principal microorganism in mVOCs production and characterization [16]. Other microorganisms include *Bacillus amyloliquefaciens* [13], *Pseudomonas fluorescens* [17], *Pseudomonas putida* [18,19], *Pseudomonas donghuensis* [20], *Streptomyces fimicarius* [21], *Trichoderma* sp. [11], etc. Thus, the mVOCs possess the potential efficacies for the replacement of chemical fertilizers and pesticides not only in field conditions but also in postharvest and storage conditions. The present review will aid the wide interdisciplinary plant biological research about mVOCs for better sustainable agricultural development.

The organic revolution in recent years has caused the increased usage of chemical inputs in augmenting sustainable agriculture. Soil infertility and multiple cropping limits have also been addressed for necessary expansions. The environmental assessment of the green revolution impact has revealed the key areas of limitations that foresee the importance of the green revolution 2.0 [22]. Further, developing countries have faced indigenous varieties in the extinction phase with intense crop practices and nutritional security [23]. Studies conducted over the last few years reveal that mVOCs have a region specificity, soil conditions, microbiome-volatile specificity, and reproducible success profiles which necessitate the arena of the green revolution 2.0 effectiveness. Thus, the utility of mVOCs and their multiple benefits are stressed for effective plant growth promotion and environmentally friendly applications for sustainable agriculture.

## 2. How mVOCs Can Have Versatile Benefits in Sustainable Agriculture?

**Figure 1:**
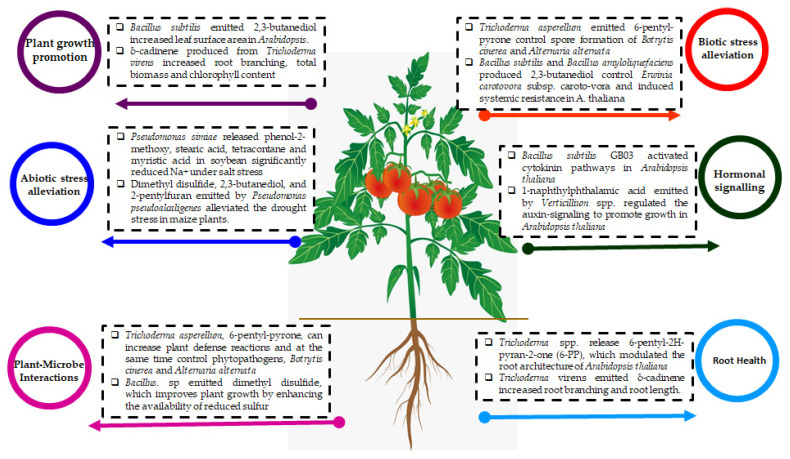
Microbial volatile organic compounds and their importance to sustainable agriculture development.

The mVOCs can offer organisms fast and precise ways to recognize neighboring organisms (both friends and foes) and to initiate specific plant growth regulation properties [1,2,3,4,24]. Recent reviews reported the wide mechanistic modes driven by mVOCs that trigger the various plant growth properties and biological activities including anti-fungal, anti-bacterial, anti-nematode, and anti-insect-pests activity, along with elicitor function representing plant immunity (both jasmonic acid and salicylic acid) [1,2,3,4,5,6,7,8,9,24,25]. Further, research on mVOCs and sustainable agriculture management has been emphasized for the controlled release of volatiles and their effective usage with the suitable strategy for plant applications [1,2,3,4,5,6,24,25]. Thus, the mVOCs have been expected as an efficient strategy for increasing plant growth, yield, defense, and productivity through a combinatorial approach for better sustainable agriculture with more benefits (Figure 1).

The mVOCs have been reported for several sustainable agricultural practices, environmentally friendly applications, phytohormones regulation, metabolic pathways signaling, and improved nutritional contents. Hence, abiotic stress mitigation, plant growth promotion, and trait improvements during plant–microbe interactions confirmed the vital qualities of mVOCs usage [11,12,13]. Several volatile organic compounds such as acetoin and associated compounds have been useful for farming practices. Further, mediators of plant growth, field application perspectives, receptor-mediated gene expression profiles, symbiosis, environmental changes, elicitor properties, and controlled release of volatiles need more research for utilization of mVOCs as an alternative for chemical fertilizers in sustainable agriculture development [24,25]. However, the present comprehensive assessment provides a deeper understanding of mVOCs, which aids plant biologists for accomplishing sustainable agriculture in farming practices.

## 3. Roles of mVOCs in Sustainable Agriculture

Plant production and food security are alarming issues in the agricultural world due to newly emerging phytopathogens and climate changes. An immediate solution for plant disease control and crop production can be accounted for with the increased use of chemical fertilizers and pesticides. However, their undue usage negatively affects both human and environmental health. Various microorganisms and their different physiological mechanisms are now being used as bioinoculants all over the world for sustainable agriculture. Several studies are being carried out to reveal new traits of microorganisms in plant production and protection [1,2,3,4,5,6,7,8,9]. Previous studies suggest that the emission of volatile organic compounds (VOCs) is one of the most predominant mechanisms by which microorganisms modulate plant growth and development [1,2,3,4,5,6,7,8,9,10,11,12,13,14].

### 3.1. The mVOCs as Plant Growth Promoters

The mVOCs can modify plant physiological and hormonal pathways to increase plant biomass and yield production via improved leaf and root characteristics, flower morphological changes, and increased fruit and seed production [26,27,28]. In 2003, Ryu et al. [29] showed that mVOCs from bacteria, *B. subtilis* GB03, increased the total leaf surface area in *Arabidopsis thaliana*. Among mVOCs, 2,3-butanediol and acetoin (3-hydroxy-2-butanone) were found to be efficient molecules for promoting plant growth, particularly shoot biomass [29,30]. Among *Bacillus* sp., *B. amyloliquefaciens* [29,30], *B. mojavensis* [31], and *B. subtilis* [29] produced 2,3-butanediol, whereas acetoin was produced by *B. amyloliquefaciens* [30] and *B. mojavensis*. Another rhizobacterium, *Serratia odorifera,* emitted a diverse and complex group of volatiles and increased the fresh weight of *A. thaliana* [32]. A study by Zou et al. [33] also showed that the mVOCs from *B. megaterium* strain XTBG34 increased the fresh weight of *A. thaliana*. Furthermore, *Bacillus* sp., isolated from rhizosphere soil of *Citrus aurantifolia,* produced the mVOCs 6,10,14-trimethyl 2-pentadecanone, benzaldehyde, and 9-octadecanone, which promoted primary root length, lateral root number, and length in *A. thaliana* [34]. Blom et al. [35] also documented that volatiles released from *Burkholderia pyrrocinia* Bcc171 elicited the increased shoot fresh weight of *A. thaliana*. Some other mVOCs from *Bacillus* sp. such as tetrahydrofuran-3-ol, 2-heptanone, and 2-ethyl-1-hexanol enhanced the plant growth in *A. thaliana* and tomato by increasing endogenous levels of auxins and strigolactones [36]. Fincheira et al. [37] used *Lactuca sativa* to investigate bacterial VOCs as a growth inducer. They found 10 bacterial strains, belonging to *Bacillus*, *Staphylococcus,* and *Serratia* genera, emitted acetoin and enhanced the plant growth by increasing the number of lateral roots, root growth, dry weight, and shoot length. The mixture of mVOCs produced by *Sinorhizobium meliloti* also promoted a significant increase in *Medicago truncatula* chlorophyll concentrations, which are an indicator of nutritional Fe status in plants. In addition, the mVOCs also induced an increase in plant biomass [38]. Another study showed the ability of soil fungi to produce mVOCs that promoted plant growth and protection [39]. Furthermore, mVOCs emitted by *P. fluorescens* elicited leaf area growth in *Mentha piperita* [40]. Park et al. [41] showed that *P. fluorescens* strain SS101 promoted tobacco growth through increased fresh weight in plants. These studies strongly suggest that mVOCs can be used as growth inducers and as an alternative or complementary strategy for application in horticulture species. 

Among mVOCs of fungal origin, in the *Trichoderma* genus there are several species whose mVOCs have been described with the ability to promote plant growth in *A. thaliana*. Contreras-Cornejo et al. [42] suggested that the mVOC δ-cadinene produced from *Trichoderma virens* increased root branching, total biomass, and chlorophyll content, whereas isobutyl alcohol, isopentyl alcohol, and 3-methylbutanal from *Trichoderma viride* accelerated flowering [43]. The mixture of mVOCs produced by *Trichoderma atroviride* enhanced the growth of *A. thaliana*, but their efficiency varied with the age of the fungal cultures [44]. In addition, mVOCs synthesized by *Alternaria alternata* modulated starch biosynthesis during illumination [45] and also increased the photosynthetic rate and accumulation of cytokinins and sugars in *A. thaliana* [46]. Schenkel et al. [47] reported that mVOCs, furfural, and 5-methyl-2-furancarboxaldehyde from *Fusarium* species increased the primary root length in *A. thaliana*. In another study, the mVOC 1-naphthylphthalamic acid from *Verticillium* species increased the auxins biosynthesis in plants [48]. Thus, both in plants and in other organisms, mVOCs can modulate the metabolome, genome, and proteome, having the undue potential to help as real biostimulants and bioprotectants, even under open-field conditions [1,49].

### 3.2. The mVOCs as a Biocontrol Agent and Plant Defense Mechanism

Biocontrol seems to be a reliable alternative to chemical fertilizers due to its eco-friendly nature and safety, which may provide long-term protection to plants. Several studies suggest that mVOCs can inhibit different types of phytopathogens and are considered a vital alternative to pesticides (Table 1). The mVOCs not only control phytopathogens, but they also increase the survival rate of microorganisms by removing potential competitors, i.e., phytopathogens for nutrients. Fernando et al. [50] showed the antifungal nature of mVOCs produced by 12 isolates of *Pseudomonas* species and their potential use in the biocontrol of phytopathogenic fungi, *Sclerotinia sclerotiorum*. Similarly, the mVOCs from two strains of endophytic *Bacillus* sp. significantly reduced the weight and number of the vegetative, long-term survival structures (sclerotia) of *S. sclerotiorum* [51]. Kai et al. [52] confirmed that rhizobacterial isolates of *P. fluorescens*, *P. trivialis, Serratia plymuthica*, *S. odorifera*, *Stenotrophomonas maltophilia*, and *S. rhizophila* produced a group of mVOCs and inhibited the growth of *Rhizoctonia solani*. *B. subtilis* emitted mVOCs such as benzaldehyde, nonanal, benzothiazole, and acetophenone, which acted against the potato ring rot causal agent, *Clavibacter michiganensis* subsp. *sepedonicus*, and reduced their colony size and other abnormalities in cells [16]. In addition, both in vitro and in vivo studies on mVOCs 2-undecanone, 2-tridecanone, and heptadecane of *B. amyloliquefaciens* not only showed biocontrol activities (inhibiting motility, biofilm formation, and root colonization) against the tomato wilt pathogen *Ralstonia solanacearum*, but also increased oxidative stress [53,54]. Xie et al. [55] proved that *Bacillus* mVOCs decyl alcohol and 3,5,5-trimethylhexanol inhibited the growth of *Xanthomonas oryzae*, the causal agent of bacterial leaf blight disease of rice. The mVOCs from *Muscodor crispans* inhibited the growth of citrus bacterial pathogen, *X. axonopodis* pv. *Citri*, and the causal agent of black sigatoka disease of banana, *Mycosphaerella fijiensis* [56]. Mycelial growth of the oomycete in *Phytophthora capsici* was inhibited significantly by VOCs (3-methyl-1-butanol, isovaleraldehyde, isovaleric acid, 2-ethylhexanol, and 2-heptanone) of *Bacillus* and *Acinetobacter* [57]. In addition, mVOCs toluene, ethyl benzene, m-xylene, and benzothiazole from *P. fluorescens* showed bacteriostatic effects [58]. Eight-carbon compounds such as 1-octen-3-ol, 3-octanol, and 3-octanone (mushroom alcohol) are among the most common fungal VOCs. Among fungal VOCs, 1-octen-3-ol inhibited the growth of phytopathogenic fungus *B. cinerea* in *A. thaliana* [59]. Kottb et al. [60] reported that the mVOC 6-pentyl-pyrone from *T. asperellum* decreased the spore formation of *B. cinerea* and *A. alternata* and enhanced the plant defense mechanisms. Few studies [61,62] reported that the VOC dimethyl disulfide, from *P. fluorescens*, *P. stutzeri,* and *Stenotrophomonas maltophilia*, gave protection against phytopathogenic fungi *B. cinerea* in tomato plants and *M. truncatula*. mVOCs such as phenyl ethanol, ethyl acetate, and methyl butanol from the yeast *Saccharomyces cerevisiae* inhibited the growth of *Guignardia citricarpa*, the causal agents of citrus black spot disease [63]. Moreover, mVOCs such as 1,3 pentadiene, acetoin, and thiophene emitted by *B. amyloliquefaciens* were effective against the post-harvest pathogens *Monilinia laxa* and *M. fructicola* in growing cherry plants as well as during their storage [64]. Zheng et al. [14] also provide evidence that mVOCs (α-farnesene) released from bacterial species (i.e., *B. pumilus*, *B. amyloliquefaciens*, and *Exiguobacterium acetylicum*) had resistance mechanisms against the post-harvest phytopathogenic fungi *Peronophythora litchi*. 

Additionally, *B. subtilis* and *B. amyloliquefaciens* released 2,3-butanediol against *Erwinia carotovora* subsp. *carotovora*-induced systemic resistance in *A. thaliana,* mediated by the ethylene-signaling pathway [65]. In tobacco, 2,3-butanediol elicited ISR against the necrotrophic bacterium *E. carotovora* subsp. *carotovora,* but not against the biotrophic bacterial pathogen *P. syringae*. Bacterial VOCs emitted from *P. polymyxa* E681 played an important role in the growth promotion and protection of *Arabidopsis* seedlings. Moreover, out of a mixture of 30 VOCs, tridecane was found to be effective against *P. syringae* pv. *maculicola* strain ES4326 via ISR mechanism [66]. Acetoin from the bacteria *B. subtilis* induced systemic resistance in *A. thaliana* against *P. syringae* through the SA-signaling pathway [67]. In maize plants, the same mVOCs from *Enterobacter aerogenes* induced resistance against the northern corn leaf blight fungus, *Setosphaeria turcica* [68]. The mVOCs from *Cladosporium* sp. showed ISR against the plant pathogen *P. syringae* [69]. *Proteus vulgaris* produced indole mVOCs, which modulated the growth of *A. thaliana* through the metabolic interplay between the auxin, cytokinin, and brassinosteroid pathways [70]. In field conditions, the effectiveness of bacterial mVOCs against bacterial diseases has been verified, as in the case of 3-pentanol emitted by *B. amyloliquefaciens*, which increases the resistance of pepper plants against bacterial spot disease (*X. axonopodis* pv. *vesicatoria*) by SA- and JA-signaling pathways [71]. *Streptomyces* spp. inhibited the production of aflatoxins from the fungal pathogen *Aspergillus flavus* through the downregulation of several genes involved in aflatoxin biosynthesis [72,73]. Exposure of *S. sclerotiorum* to mVOCs produced by *Trichoderma* species led to the upregulation of four glutathione S-transferase genes, which are involved in the detoxification of antifungal secondary metabolites, which may contribute to the virulence of *S. sclerotiorum* [74]. The activation of plant defenses by mVOCs has been extensively studied, including in vitro and even in field assays. Future lines of research should be carried out to develop formulations and methodologies for direct use in agriculture.

### 3.3. mVOCs as an Abiotic Stress Ameliorator

Apart from biotic stress alleviation, mVOCs also increased abiotic stress tolerance in plants, but limited studies have been documented to date. Zhang et al. [75] showed that *B. subtilis* emitted 2, 3-butanediol contributed salt tolerance in *Arabidopsis* and downregulated expression of K^+^ transporter 1 in roots with upregulation in shoots of *A. thaliana*. The regulation in expression (upregulation vs downregulation) helped the regulation of Na^+^ accumulation and, henceforth, enhanced tolerance to salt stress. In addition, salt-stressed *Arabidopsis* plants treated with *B. subtilis* GB03 VOCs showed greater biomass production and less Na^+^ accumulation compared to salt-stressed plants. Whereas the same compound produced by *P. chlororaphis* resulted in drought tolerance, which resulted from increased stomatal closure and reduced water loss [76]. In a subsequent study, 2,3-butanediol was found to induce plant production of nitric oxide (NO) and hydrogen peroxide, while chemical perturbation of NO accumulation impaired 2,3-butanediol-stimulated plant survival under drought stress. The above results indicated an important role for NO signaling in the drought tolerance induced by 2,3-butanediol [77]. Under osmotic stress, *Arabidopsis* exposed to GB03 VOCs accumulated higher levels of choline and glycine betaine than plants without VOC treatment [78]. Li and Kang, [12] proved that mVOCs from *Verticillium dahliae* increased defense signaling against salt stress by auxins in *A. thaliana*. According to del Rosario-Cappellari and Banchio [13], acetoin emitted by *B. amyloliquefaciens* on *M. piperita* showed increased tolerance to salinity and also increased chlorophyll and salicylic acid contents. In addition, *P. simiae* released mVOCs phenol-2-methoxy, stearic acid, tetracontane, and myristic acid in soybean, which significantly reduced Na^+^ and increased K^+^ and P uptake in roots under salt stress, which is also due to upregulation of peroxidase, catalase, vegetative storage protein, and nitrite reductase genes [79,80]. The emissions not only decreased root Na+ levels but also increased the accumulation of proline, which protects cells from osmotic stress [80]. *B. thuringiensis* AZP2 and *Paenibacillus polymyxa* B emitted three volatile compounds, benzaldehyde, β-pinene, and geranyl acetone, in wheat seeds, which showed enhanced tolerance against drought stress and also showed increased dry weight, water use efficiency, and antioxidant enzyme activity [81]. According to Ledger et al. [82], mVOCs 2-undecanone, 7-hexanol, 3-methylbutanol, and dimethyl disulfide emitted from *Paraburkholderia phytofirmans* PsJN showed increasing plant growth rate and tolerance to salinity. Interestingly, Yasmin et al. [83] reported that mVOCs dimethyl disulfide, 2,3-butanediol, and 2-pentylfuran emitted by *P. pseudoalcaligenes* alleviated the drought stress in maize plants. Li et al. [84] concluded that *Rahnella aquatilis* JZ-GX1 VOCs (2,3-butanediol) had a significant plant growth-promoting effect on *Robinia pseudoacacia* seedlings under salt stress conditions. Importantly, the sodium-potassium ratios in the roots, stems, and leaves of acacia exposed to VOCs of the JZ-GX1 strain were significantly lower than those in the control samples. The capacity of mVOCs to upsurge plant tolerance to abiotic stresses such as salinity and drought has been reported in plants. However, the exact application of mVOCs in the agriculture field to increase the productivity of crops under abiotic stress conditions needs further studies.

### 3.4. mVOCs Modulate Plant Hormonal Signaling

Some mVOCs were proven to modulate plant growth by modifying the biosynthesis, perception, and homeostasis of the plant hormones. Plant growth modification corresponds to the modulation of the salicylic acid, jasmonic acid/ethylene, and auxin signaling pathways. Several studies with *A. thaliana* have shown vital signs that mVOCs can modulate phytohormone pathways. Ryu et al. [29] reported that mVOCs released by *B. subtilis* GB03 activated cytokinin pathways in *A. thaliana*, playing an important role in the surface area of the leaf. In addition, auxin homeostasis in *A. thaliana* was modulated by mVOCs of *B. subtilis* GB03, whereas genes of auxin biosynthesis (*NIT1* and *NIT2*) and responsive genes were up-regulated [85]. Bailly et al. [86] proved that lateral root development in *A. thaliana* was regulated by indole released by *Escherichia coli* through modulation of the auxin signaling pathway. In addition, mVOCs emitted from *P. vulgaris* JBLS202 regulated different pathways such as cytokinin, brassinosteroid, and auxin pathways for the growth of *A. thaliana* [70]. *Trichoderma* spp. released 6-pentyl-2H-pyran-2-one (6-PP), which modulated the root architecture of *A. thaliana* by auxin signaling pathways through the modulation of PIN-auxin transport proteins in specific root tissue. 6-PP modulated the function of auxin receptors (*TIR1*, *AFB2*, and *AFB3*), influencing lateral root development [87]. Moreover, it was reported that VOCs emitted by *A. alternata* stimulated the accumulation of cytokinin, which played an important role in the growth of *A. thaliana* [46]. In addition, mVOCs emitted from *B. methylotrophicus* M4–96 promoted the enhanced concentration of indole acetic acid in the shoot and root of *A. thaliana*, indicating that the activation of the auxin pathway increased the auxin content in *A. thaliana* [88]. According to Zhou et al. [89], mVOCs emitted from *B. amyloliquefaciens* strain SAY09 alleviated the cadmium toxicity in *A. thaliana* via enhanced auxin biosynthesis. Further, mVOCs emitted by *B. subtilis* SYST2 increased the concentration of auxin and cytokinin in *S. lycopersicum* seedlings, which was supported by the up-regulation of genes related to their biosynthesis [90]. Recently, Jiang et al. [36] reported that *A. thaliana* growth was enhanced through auxin and strigolactone action by VOCs released from *Bacillus* sp. JC03. Moreover, VOCs emitted by *R. solani* up-regulated genes associated with auxin (*IAA-2*, *IAA-19*, *IAA-29*, *PIF5*, and *HB-2*) and abscisic acid (*CYP707A43*) pathways in *A. thaliana* [91]. Interestingly, the mVOC 1-naphthylphthalamic acid emitted by *Verticillium* spp. regulated the auxin signaling to promote growth in *A. thaliana*, which was noted in the mutants (*AUX1*, *TIR1*, and *AXR1*) [48]. Finally, it is noted that VOCs emitted by *F. luteovirens* increased the lateral root number in *A. thaliana* and reduced the auxin accumulation in primary root length through the repression of auxin efflux carrier PIN-FORMED 2 (*PIN2*) [92]. Thus, mVOCs contribute significantly to the regulation of many crucial signaling and physiological processes and enhance the overall growth and vigor of plants.

## 4. mVOCs on Intra and Inter-Species Interactions

Microbial interaction plays an important role within and outside kingdom interaction due to a variety of compounds and secondary metabolites released by several microorganisms. The various functions of mVOCs correspond to the modulation of microbe–microbe and microbe–plant interactions via signaling molecules, which regulate the key physiological processes [9,10,11,12,13,14,15,16,17,49,93,94,95]. The mVOCs produced belong to several classes (ketones, alcohols, pyrazines, alkenes, sulfides, benzenoids, terpenes, etc.). The mVOCs production is influenced by various factors including microbial growth stage, availability of nutrients, oxygen and moisture contents, pH, temperature, etc. [4]. mVOC-producing microorganisms not only communicated with other organisms but also increased their survival efficiency, which enabled them to specific and evolutionary-associated mVOC traits. At the same time, communicating (micro) organisms can develop physiological mechanisms for mVOC perception and tolerance. Hence, mVOCs act as mediators of ecological intra- and interspecific interactions, ranging from microbe–microbe communication to cross-domain interactions.

The mVOCs have been proven for various environmental adaptations of within/intra species interaction and modulation of the biochemical properties. The inherent changes can be attributed to changes in pH, disruption of quorum sensing, and regulation of phytopathogenicity (e.g., virulence protein production). Jones et al. [96] showed that *Streptomyces venezuelae* synthesized trimethylamine upon increased pH in their medium, which in turn reduced the availability of local iron in the niche. The mixture of mVOCs 1-undecene, methyl thiolacetate, and dimethyl disulfide from *Pseudomonas chlororaphis* reduced the quorum-sensing signals required for phenazine biosynthesis and also suppressed the expression of N-acyl-homoserine lactones biosynthetic genes [97]. Moreover, the mVOCs leudiazen can regulate the production of mangotoxin in *Pseudomonas syringae* pv. *syringae* [98]. Further, the mVOCs can regulate their phytopathogenicity and act as an antimicrobial substance, facilitating colonization of the phyllosphere by *P. syringae* pv. *syringae*.

The wide diversity of mVOCs mediates complex and yet unknown interactions between and inter-kingdom, and these attributes emphasize the importance of specificity and cross-reactivity of mVOCs and their evolutionary significance [4,25,28]. For example, geosmin, generally identified in soils, has been linked to olfactory receptors in insects by the interaction between the taxonomically distant organisms *Streptomyces* and the soil arthropod *Folsomia candida* [99]. Few studies have shown that the production of geosmin and 2- methylisoborneol by *Streptomyces* attracts *F. candida* [99,100,101]. In these inter-kingdom interactions, *F. candida* supports the dispersal of bacterial spores via feeding and attachment to their cuticle, which, in turn, are benefited through reproductive success such as higher arthropod molting and egg laying. Another study showed that an array of mVOCs (including decanal, 2-ethylhexyl acetate, 3,5-dimethylbenzaldehyde, and ethyl acetate) emitted by the bacteria *Listeria monocytogenes* attract the protozoan *Euglena gracilis* in soil [102]. This protozoan feeds on specific bacterial taxa, imposing a strong selective pressure by favoring the persistence and evolution of adaptive traits to resist predation [103]. There is a decrease in spore formation of *B. cinerea* and *A. alternata*, and an increase in plant defense reactions is due to a 6-pentyl-pyrone, a distinguishing compound of *T. asperellum* [60]. mVOCs also facilitate the bidirectional inter-species communications between *Verticillium longisporum*- *P. polymyxa* [104] and *Aspergillus flavus*- *Ralstonia solanacearum* [105]. *B. amyloliquefaciens* emitted the mVOCs pyrazine and 2,5-dimethylpyrazine, eliciting both jasmonic acid and salicylic acid pathway-mediated defense in the phyllosphere of plants [106]. Moreover, the ability of bacteria and fungi to communicate with each other is a remarkable aspect of the microbial world. Schmidt et al. [107] performed transcriptomics and proteomics analyses of the bacterium *Serratia plymuthica* exposed to VOCs emitted by the fungal pathogen *Fusarium culmorum*. They found that the bacterium responded to fungal VOCs and changed their gene and protein expression related to motility, signal transduction, energy metabolism, cell envelope biogenesis, and secondary metabolite production. Hence, the ecology and adaptation of microorganisms and their mVOCs account for the assessment of the mechanism of eco-evolutionary dynamics and thereby determine the specificity and cross-reactivity of mVOCs.

## 5. Recent Research on mVOCs

Plant and soil-associated microorganisms release a wide variety of mVOCs, which was reported (Table 2); however, even today, the ecological and physiological functions of many mVOCs are not understood in detail and require further research. Volatile organic compounds released by endophytic bacteria comprising *Acinetobacter*, *Arthrobacter*, *Bacillus*, *Microbacterium*, *Pantoea*, *Pseudomonas*, and *Stenotrophomonas* sp. inhibited the growth of fungal pathogens, namely *Alternaria alternata* and *Corynespora cassiicola* [108]. Recent studies have shown the ability of soil fungi to produce mVOCs that enhance plant growth and protection [39,109,110,111]. Velásquez et al. [39] showed the difference in patterns of mVOC production during the interaction of arbuscular mycorrhizal fungus *Funneliformis mosseae* and plant growth promoting rhizobacterium *Ensifer meliloti,* which specified the apparent roles in sustainable vineyard management. In this study, monoterpenes were strongly enhanced by *F. mosseae* by increased plant defense, whereas *E. meliloti* did not significantly affect mVOC production and defense. There are beneficial effects of *Trichoderma* strains found in root ecosystems and soil to enhance plant growth by mVOCs. An mVOC released from endophytic fungi, *Trichoderma asperellum,* revealed potent antifungal activity against leaf spot pathogens *Corynespora cassiicola* and *Curvularia aeria*, with plant growth promotion in lettuce [112]. Moreover, mVOCs have also been documented for endophytic *Trichoderma* spp.- *Sclerotinia sclerotiorum*, *Sclerotium rolfsii,* and *Fusarium oxysporum* interaction through mycoparasitism [113]. An earlier study indicated that mVOCs from non-pathogenic *F. oxysporum* were found to be effective in combating *Verticillium* wilt, revealing the importance of non-pathogenic species for upcoming plant protection strategies [114]. Recently, Junior et al. [115] showed the importance of mVOCs as an alternative to reduce the use of traditional synthetic fungicides. They found that the yeast *Starmerella bacillaris* synthesized volatile organic compounds, which showed a reduction of apple gray mold (*B. cinerea*) disease and regulated cider aromatic qualitative profiles along with antimicrobial biocontrol activities mediated by benzyl alcohol. In addition to plant growth promotion and plant protection, post-harvest disease control has also been accounted for in the advanced benefits of mVOCs. *Aureobasidium pullulansi* L1 and L8 strains revealed antagonistic activities against two yeast strains, *Monilinia fructigena* and *M. fructicola,* in combating brown rot disease in stone rot fruits in post-harvest control [116]. A recent study proved the effective biocontrol of post-harvest litchi fruit pathogen *P. litchii* by mVOCs. They found that benzothiazole had an antagonist effect against *P. litchii*, whereas α-farnesene might induce plant defense mechanisms [14,21]. Many fungal VOCs are found to be identical to natural flavorings and fragrances produced by plant molecules and are therefore of huge importance in the chemical, feed, pharmaceutical, food, and cosmetic industries.

Morita et al. [117] proved that *Bacillus pumilus* emitted mVOCs, particularly methyl isobutyl ketone, ethanol, 5-methyl-2-heptanone, and S-2-methylbutylamine, that had antifungal spectrum effects against food-deteriorating fungi during storage. Further, endophytic *Pseudomonas putida* BP25 from black pepper was proven as an environmentally-friendly approach to combating oomycete pathogens (*Phytophthora capsici* and *Pythium myriotylum*), fungal pathogens (*Rhizoctonia solani*, *Colletotrichum gloeosporioides*, *Athelia rolfsii*, *Gibberella moniliformis*, and *Magnaporthe oryzae*), bacterial pathogens (*Ralstonia pseudosolanacearum*), and plant parasitic nematodes (*Radopholus similis*) [19]. Benzoic acid ethyl ester, 3-methyl-butanoic acid and 2-ethyl-1-hexanol were the inherent volatile organic compounds present in the rhizosphere region of rice elicited by *R. solani*, the usual rice sheath blight pathogen [118]. *Bacillus* spp. in the avocado rhizosphere synthesized volatile organic compounds including ketones, pyrazines, and sulfur-containing compounds for arresting dieback disease caused by *Fusarium* sp. [119]. *B. subtilis* CF-3 secreted volatile organic compounds including 2,4-di-tert-butylthiophenol and benzothiazole, showing potential anti-fungal activities against *Colletotrichum gloeosporioides* and *Monilinia fructicola,* thus hindering fermentation [120]. *B. subtilis* CF-3 secreted volatile organic compounds in combating *Monilinia fructicola* through the activation of disease-resistant enzymes encompassing phenylalanine ammonia-lyase, chitinases, and β-1,3-glucanase in peaches [121]. Volatile organic compounds produced by *B. velezensis* CT32 showed anti-fungal and biofumigation properties against *Verticillium dahliae* and *F. oxysporum,* causing vascular wilt [122]. Thus, broad-spectrum anti-microbial activities affirm the efficacy of mVOCs [19]. The research establishes that mVOCs secreted by microorganisms in plant ecosystems could have prominent implications in beneficial soil resources contributing to soil and plant health.

Distinct fumigation activity and anti-fungal potentials were affirmed from *Streptomyces* sp. strain S97-derived volatile organic compounds controlling *B. cinerea* in strawberries [123]. *Streptomyces yanglinensis* 3–10 produced volatile organic compounds that possess fumigation potentials in curbing *A. flavus* and *A. parasiticus,* causing contamination of the peanut kernel storage environment [73]. Biocontrol yeasts comprising *Wickerhamomyces anomalus*, *Metschnikowia pulcherrima*, *Aureobasidium pullulans,* and *Saccharomyces cerevisiae*-derived volatile organic compounds revealed biocontrol efficiency and synergy with carbon dioxide to prevent post-harvest loss in packaging scenarios [124]. Meloidogyne-based disease complexes have been linked to volatile organic compounds of biocontrol nematicidal agents [125]. To repel banana weevil pests, *Cosmopolites sordidus* was arrested using volatile organic compounds synthesized from entomopathogenic fungi, *Beauveria bassiana* (Bb1TS11) and *Metarhizium robertsii* (Mr4TS04) [126]. The 1-undecene derived from plant growth promoting *Pseudomonas* sp. ST–TJ4 revealed the effective volatile organic compounds in sustainable management of agroforestry ecosystems against various phytopathogenic fungi [127]. Volatile organic compounds have been also proven effective in deciphering insect–microbe symbiotic association in the spruce bark beetle; *Ips typographus* shows forest pest management strategies [128]. Further, the replacement of chemical fertilizers and pesticides by volatile organic compounds derived from microbial sources for combating the rise in population and demand in the food supply, and ensuring sustainable agriculture [1,15,25,28,93]. The research scenario updates over the past five years depict the advanced methodologies and strategies for using volatile organic compounds in sustainable agriculture management. Hence, a cataloging project involving the assessment of the pros and cons of mVOCs is needed. Further, the intricate studies for deciphering the mode of action, specificity, and sensitivity of mVOCs in sustainable agriculture are required for improvising sustainable agriculture development goals for a better future.

**Table 2:** Microbial volatile organic compounds and their potential biological roles.

S.No.	Microbial Volatile Organic Compounds Produce Microorganisms	Biological Roles	References
1.	*Acinetobacter*, *Arthrobacter*, *Bacillus*, *Microbacterium*, *Pantoea*, *Pseudomonas*, and *Stenotrophomonas* sp.	Antifungal activity against *Alternaria alternata* and *Corynespora cassiicola*	[108]
2.	*Funneliformis mosseae*(AMF)–*Ensifer meliloti* (Rhizobacterium) interaction	Sustainable vineyard management	[39]
3.	*Bacillus* sp. JC03	Plant growth promotion in *Arabidopsis thaliana,*	[36]
4.	*Aureobasidium pullulansi* L1 and L8	*Monilinia fructigena,* and *Monilinia fructicola* yeast Antagonism and post-harvest brown rot control	[116]
5.	*Starmerella bacillaris*	Apple gray mold disease control and the rich aroma of cider through benzyl alcohol	[115]
6.	Endophytic fungus in *Trichoderma asperellum* T1	Antifungal activity against *Corynespora cassiicola* and *Curvularia aeria,* plant growth promotion and defense mechanisms	[112]
7.	*Bacillus pumilus* TM-R	Wide antifungal activity	[117]
8.	Endophytic *Trichoderma* spp- *Sclerotinia sclerotiorum-*TSS*, Sclerotium rolfsii*-CSR, and *Fusarium oxysporum*-CFO interaction	Mycoparasitic activity	[113]
9.	Endophytic *Pseudomonas putida* BP25	Broad spectrum activity against oomycete pathogens (*Phytophthora capsici* and *Pythium myriotylum),* fungal pathogens (*Rhizoctonia solani*, *Colletotrichum gloeosporioides*, *Athelia rolfsii*, *Gibberella moniliformis* and *Magnaporthe oryzae*), bacterial pathogens (*Ralstonia pseudosolanacearum*), and plant parasitic nematodes (*Radopholus similis)*	[19]
10.	Rhizospheric *Rhizoctonia solani* (pathogenic)	Beneficial soil and plant health	[118]
11.	Non-pathogenic *Fusarium oxysporum* FO12	Verticillium wilt abatement	[114]
12.	*Streptomyces* sp. strain S97	*Botrytis cinerea* control in strawberry	[123]
13.	*Bacillus* spp. in avocado rhizosphere	Dieback disease due to *Fusarium* sp.	[119]
15.	*Bacillus subtilis* CF-3	Antifungal activity against *Colletotrichum gloeosporioides* and *Monilinia fructicola*	[120]
16.	*Streptomyces yanglinensis* 3–10	Control of *Aspergillus flavus* and *Aspergillus parasiticus* in peanut kernel storage	[73]
17.	Entomoptahogenic fungi *Beauveria bassiana* (Bb1TS11) and *Metarhizium robertsii* (Mr4TS04)	The arrest of repelling banana weevil pests, *Cosmopolites sordidus*	[126]
18.	*Pseudomonas* sp. ST–TJ4	Wide spectrum phytopathogenic activity in agroforestry	[19]
19.	*Wickerhamomyces anomalus*, *Metschnikowia pulcherrima*, *Aureobasidium pullulans,* and *Saccharomyces cerevisiae* (Biocontrol yeasts)	Biocontrol agents and carbon dioxide synergy for prevention of post-harvest loss	[124]
20.	*Bacillus velezensis* CT32	Biofumigation activities against *Verticillium dahliae* and *Fusarium oxysporum,* vascular wilt pathogens	[122]
21.	Insect–microbe symbiosis in Spruce bark beetle, *Ips typographus*	Forest pest management	[128]

## 6. Environmental Friendliness and Limitations of mVOCs

**Figure 2:**
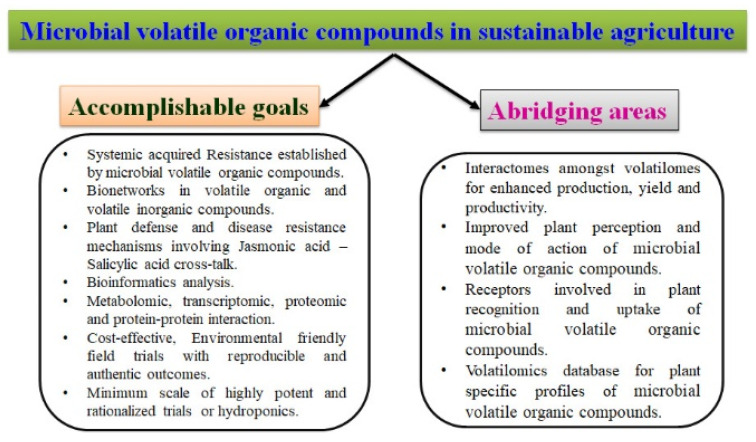
Goals and abridging areas of sustainable agriculture.

The discussion on mVOCs synthesized from microorganism and their interactions confirmed the efficacy and specificity of mVOCs for sustainable agriculture and development. Nevertheless, mVOCs have been focused on for better management of plant growth. The inherent mechanisms include plant growth regulation, inhibition of phytopathogens, priming plant defense signals, induction of plant defense, hormone-mediated plant homeostasis, anti-microbial efficacies, etc. [1,2,25,28,93,94]. Thus, the realm of mVOCs poses intricate and intensive research for deriving the volatile organic compound applications and their potential interactions in ascertaining sustainable agriculture (Figure 2).

In addition, endophytic fungal-derived mVOCs have been reviewed extensively for their anti-bacterial and anti-fungal properties, ascertaining the phytotoxicity implications [56,113,129,130,131]. Nonetheless, the detoxification strategies employed by the mVOCs will have potentiating benefits in instituting sustainable agriculture [129,130,131,132]. The detoxification potentials of mVOCs have been extensively reported by plant biologists and long-term soil fertility has been reported in vineyard soils (viticulture) [130]. Moreover, volatile organic compounds in plant–microbe interactions will lead to successive field trials after intricate molecular studies on plant perception of volatiles, receptor-mediated endocytosis mechanisms, and differential profiling of volatiles [132]. Further, post-harvest loss diseases have been addressed through biofumigation with mVOCs in effective management [133,134,135,136,137,138]. Thus, versatile benefits of microbial-based volatile organic compounds are summarized for better agriculture environmental friendliness and sustainability.

Various environmental factors such as microbial growth conditions, microbial community, availability of nutrients, and oxygen, temperature, and pH influence the production of mVOCs [104,105,106,107]. These environmental factors made it difficult to identify whether the effect was on an individual molecule and what was the mechanism. Hence, the commercial application of these volatiles is very limited compared to the economic implications. In addition, there are varying differences in volatile compound effects from lab to field. The non-reproducibility of results, authenticity, and cost-effective applications predict the application of smart agricultural practices. The incorporation of big data computing analytics, phenotyping, and sensors for continuous monitoring of volatile organic compounds is stressed for sustainable agriculture [105,106,107]. Therefore, the limitations addressed in the applicability of volatiles can be rectified using cost-effective technological advancements and environmentally friendly management approaches.

## 7. Concluding Remarks and Future Perspectives

The mVOCs research in plant–microbe interactions, microbe–microbe interactions, and respective positive benefits in sustainable agriculture and plant productivity has been elaborated for future advancements. Studies about plant growth promotion, plant defense, stress tolerance, and ISR mechanisms indicate the significance of mVOCs and their vital role in sustainable agriculture. Further, the lab-to-field transition of experiments involving the perception of volatiles, interacting pathways, and gene regulation has been fortified for more sustainable agricultural practices. Environmentally friendly, cost-effective field trials, novel incorporations for hydroponics, and volatile success rates can open a new avenue of sustainability research. Further, volatiles will contribute to the majority of research prospects in the green tevolution 2.0 for surpassing large-scale chemical fertilizers and pesticide usage with increased organic farming inputs for agricultural nutrition. Even though the efficacy of different mVOCs in plant–microbe interactions has been widely studied, the precise mechanisms involved are still unknown. This signifies the importance of more research on mVOCs and plant–microbe interaction studies. Hence, future perspectives will hold promising multi-omics, big data analytics, and biosensor technology in authenticating physiological mechanisms and the field trial’s success of volatiles, especially mVOCs.

## Figures and Tables

**Table 1:** Summary of microbial volatile compounds and their plant disease control.

Microorganism	Microbial Volatile Compounds	Controlled Plant Pathogen	References
*Pseudomonas fluorescens* *Pseudomonas corrugate* *Pseudomonas chlororaphis* *Pseudomonas aurantiaca*	Benzothiazole Cyclohexanol *n*-Decanal Dimethyl trisulfide 2-Ethyl 1-hexanol Nonanal	*Sclerotinia sclerotiorum*	[50]
*Bacillus velezensis*	Benzothiazole	*Sclerotinia sclerotiorum*	[51]
*Pseudomonas fluorescens* *Pseudomonas trivialis* *Serratia plymuthica* *Serratia odorifera* *Stenotrophomonas maltophilia* *Stenotrophomonas rhizophila*	β-Phenylethanol Dimethyl trisulfide	*Rhizoctonia solani*	[52]
*Bacillus subtilis*	Benzaldehyde Nonanal Benzothiazole Acetophenone	*Clavibacter michiganensis* sp. *sepedonicus*	[16]
*Bacillus amyloliquefaciens*	2-Undecanone 2-Tridecanone Heptadecane	*Ralstonia solanacearum*	[53,54]
*Bacillus* strain D13	Decyl alcohol 3,5,5-Trimethylhexanol	*Xanthomonas oryzae*	[55]
*Muscodor crispans*	Propanoic acid 2-Methyl- compounds	*Pythium ultimum* *Phytophthora cinnamom* *Sclerotinia sclerotiorum* *Mycosphaerella fijiensis* *Xanthomonas axonopodis* pv. *citri*	[56]
*Bacillus* and *Acinetobacter*	3-Methyl-1-Butanol Isovaleraldehyde Isovaleric acid 2-Ethylhexanol 2-Heptanone	*Phytophthora capsici*	[57]
*Pseudomonas fluorescens* WR-1	Toluene, Ethyl benzene, m-Xylene Benzothiazole	*Ralstonia solanacearum*	[58]
*Penicillium glabrum*	1-Octen-3-ol	*Botrytis cinerea*	[59]
*Trichoderma asperellum*	6-Pentyl-pyrone	*Botrytis cinerea* *Alternaria alternata*	[60]
*Pseudomonas fluorescens* *Pseudomonas stutzeri* *Stenotrophomonas maltophilia*	Dimethyldisulfide	*Botrytis cinerea*	[61,62]
*Saccharomyces cerevisiae*	Phenyl Ethanol Ethyl acetate Methylbutanol	*Guignardia citricarpa*	[63]
*Bacillus amyloliquefaciens*	1-(2-Aminophenyl) Ethanone Benzothiazole	*Peronophythora litchii*	[14]
*Bacillus amyloliquefaciens*	1,3 Pentadiene Acetoin Thiophene	*Monilinia laxa* *Monilinia fructicola*	[64]
*Bacillus subtilis* *Bacillus amyloliquefaciens*	2,3-Butanediol	*Erwinia carotovora* subsp. *carotovora*	[65]
*Paenibacillus polymyxa*	Tridecane	*Pseudomonas syringae* pv. *maculicola*	[66]
*Enterobacter aerogenes*	Acetoin	*Setosphaeria turcica*	[67]
*Bacillus subtilis*	acetoin (3-hydroxy-2-butanone)	*Pseudomonas syringae pv. tomato DC3000*	[68]
*Ampelomyces* sp. and *Cladosporium* sp.	m-cresol and methyl benzoate	*Pseudomonas syringae pv. tomato DC3000*	[69]
*Proteus vulgaris JBLS202*	Indole	*Plant hormone signaling pathway*	[70]
*Bacillus amyloliquefaciens*	3-Pentanol	*Xanthomonas axonopodis* pv. *vesicatoria*	[71]
*Streptomyces alboflavus* TD-1	Dimethyl trisulfide Benzenamine	*Aspergillus flavus*	[72]
*Streptomyces yanglinensis* 3–10	2-Methylbutyrate 2-Phenylethanol β-Caryophyllene	*Aspergillus flavus* *Aspergillus parasiticus*	[73]

## Data Availability

Not applicable.
